# Voluntary exercise modulates pathways associated with amelioration of retinal degenerative diseases

**DOI:** 10.3389/fphys.2023.1116898

**Published:** 2023-03-10

**Authors:** Joshua A. Chu-Tan, Adrian V. Cioanca, Yvette Wooff, Max Kirkby, Marissa Ellis, Pranay Gulati, Tim Karl, Jeffrey H. Boatright, Katie Bales, John Nickerson, Riccardo Natoli

**Affiliations:** ^1^ Eccles Institute of Neuroscience, John Curtin School of Medical Research, College of Health and Medicine, The Australian National University, Acton, ACT, Australia; ^2^ School of Medicine and Psychology, College of Health and Medicine, The Australian National University, Acton, ACT, Australia; ^3^ School of Medicine, Western Sydney University, Penrith, NSW, Australia; ^4^ Department of Ophthalmology, Emory University, Atlanta, GA, United States

**Keywords:** retina, exercise, inflammation, retinal degeneration, cellular protection, age-related macular degeneration

## Abstract

**Background:** Exercise has been shown to promote a healthier and longer life and linked to a reduced risk of developing neurodegenerative diseases including retinal degenerations. However, the molecular pathways underpinning exercise-induced cellular protection are not well understood. In this work we aim to profile the molecular changes underlying exercise-induced retinal protection and investigate how exercise-induced inflammatory pathway modulation may slow the progression of retinal degenerations.

**Methods:** Female C57Bl/6J mice at 6 weeks old were given free access to open voluntary running wheels for a period of 28 days and then subjected to 5 days of photo-oxidative damage (PD)-induced retinal degeneration. Following, retinal function (electroretinography; ERG), morphology (optical coherence tomography; OCT) and measures of cell death (TUNEL) and inflammation (IBA1) were analysed and compared to sedentary controls. To decipher global gene expression changes as a result of voluntary exercise, RNA sequencing and pathway and modular gene co-expression analyses were performed on retinal lysates of exercised and sedentary mice that were subjected to PD, as well as healthy dim-reared controls.

**Results:** Following 5 days of PD, exercised mice had significantly preserved retinal function, integrity and reduced levels of retinal cell death and inflammation, compared to sedentary controls. In response to voluntary exercise, inflammatory and extracellular matrix integrity pathways were significantly modulated, with the gene expression profile of exercised mice more closely trending towards that of a healthy dim-reared retina.

**Conclusion:** We suggest that voluntary exercise may mediate retinal protection by influencing key pathways involved in regulating retinal health and shifting the transcriptomic profile to a healthy phenotype.

## Background

Physical activity and exercise are synonymous with health and longevity. The benefits of exercise to the human body are well-established, with overwhelming evidence demonstrating amelioration of both physical and mental aspects of human physiology (Pedersen and Saltin, 2015). Exercise has been shown to improve the pathology of several chronic diseases including cardiovascular disease, Type-2 diabetes, obesity, and cancer (Pedersen and Saltin, 2015). In addition, as a modifiable risk factor alongside diet, physical activity has more recently been considered to be an important alternative therapeutic intervention to slow cognitive decline and pathophysiological processes in age-related neurological conditions and neurodegenerative disorders, including in Parkinson’s (Chen et al., 2005; Xu et al., 2010) and Alzheimer’s diseases (Buchman et al., 2012), as well as improving memory function in adults over the age of 65 (Colcombe and Kramer, 2003; Larson et al., 2006).

The broad beneficial effects of exercise observed across disorders of the central nervous system (CNS), have more recently been demonstrated in the retina, with strong evidence emerging that exercise offers protection against retinal degenerations, including in glaucoma (Williams, 2009a; Chrysostomou et al., 2014; Meier et al., 2018), diabetic retinopathy (Allen et al., 2018), retinitis pigmentosa (Hanif et al., 2015) and in age-related macular degeneration (AMD) (McGuinness et al., 2017; Mees et al., 2019).

AMD, the leading cause of blindness in the developed world (Deloitte Access Economics and Mitchell, 2011), is a chronic and progressive disease characterised by overburdening levels of inflammation, consequently resulting in the death of the light-sensing photoreceptor cells and irreversible blindness (Kauppinen et al., 2016). Exercise has been shown to reduce the severity of AMD, with higher levels of vigorous exercise in middle-aged adults (Williams, 2009b) and moderate exercise in people over 75 associated with a lower incidence rate of AMD (Gopinath et al., 2014). Conversely, decreased physical activity was associated with an increase in early pathogenic features, or “precursors” of AMD (Munch et al., 2013).

It has been suggested that one of the main mechanisms of action for the health benefits of exercise in neurodegenerative diseases is through modulating the activation of glia leading to reduced pro-inflammatory cytokines and neuroinflammation (Seo et al., 2019). As neuroinflammation is a hallmark feature of neuro- and retinal degenerative diseases such as AMD, targeting, or influencing endogenous anti-inflammatory mechanisms are of considerable interest in therapeutic development (Kauppinen et al., 2016). Given that AMD is projected to affect 1.7 million Australians by 2030, and 288 million worldwide by 2040 (Deloitte Access Economics and Mitchell, 2011; Wong et al., 2014), any treatments which could slow the progression of this disease, and others where underlying neuroinflammation is a key pathological feature, would greatly reduce the significant economic impacts and have major impacts on an individual’s quality of life.

Research has shown that exercise is able to regulate systemic inflammation, by modulating key inflammatory pathways and pro-inflammatory cytokine profiles that lead to downstream dampening of innate immune responses (Nieman and Wentz, 2019; Chu‐Tan et al., 2021). In the retina, inflammatory modulation in response to exercise has been correlated with protection against degeneration (Chrysostomou et al., 2016; Mees et al., 2019; Dantis Pereira de Campos et al., 2020; Bales et al., 2022). In rodent models of retinitis pigmentosa, voluntary exercise has been shown to be effective against photoreceptor cell loss and inflammation as well as increase visual acuity (Barone et al., 2012; Barone et al., 2014; Zhang et al., 2019), while swimming used in a model of glaucoma was protected against astrocytic gliosis, macrophage activation and age-related vulnerability (Chrysostomou et al., 2014). Furthermore, treadmill exercise was shown to protect visual function, and decrease inflammation and apoptotic neuronal cell death in a model of diabetic retinopathy (Ji et al., 2013; Kim et al., 2013; Allen et al., 2018; Dantis Pereira de Campos et al., 2020). Finally, in a light-induced model of degeneration that recapitulates aspects of atrophic AMD (Natoli et al., 2016), forced treadmill exercise in albino mice was shown to preserve both retinal morphology and function (Lawson et al., 2014; Mees et al., 2019) with low-intensity exercise proving the most beneficial (Mees et al., 2019). Additionally, this model also revealed retinal astrocytes have altered morphology and increased expression of brain-derived neurotrophic factor (BDNF) and a specific isoform of its high-affinity receptor TrkB (Bales et al., 2022).

Although these studies collectively shed light on the therapeutic potential of exercise to reduce inflammation and protect against, or slow the progression of, retinal degenerations, knowledge on the precise molecular events occurring within the retina during exercise is still lacking. Clinical research has largely been confined to correlative epidemiological studies, whereas animal studies have been limited to implicative processes and/or based on single, select molecular targets (Chu‐Tan et al., 2021). Further, as animal studies to date have predominantly employed a forced model of exercise, it is unclear what potentially confounding effects stress induced shock-forcing methods may have on the results of these studies, with known stress and anxiety-like behaviours exhibited in forced vs. voluntary-exercise animals (Leasure and Jones, 2008) as well as stress having a known impact on inflammation in the retina (Malan et al., 2020).

Therefore, to address these highlighted problems and help decipher the molecular effect of physical activity on retinal protection, we have employed a voluntary rodent model of aerobic exercise using open voluntary running wheels, and a well-established photo-oxidative damage (PD) model of retinal degeneration that recapitulates key inflammatory aspects of AMD (Natoli et al., 2016). We determined that voluntary exercise provides protection to the retina against photo-oxidative damage with both functional, morphological and histological improvements, in particular in reducing photoreceptor cell death and inflammation. Further, RNA sequencing and gene module co-expression analyses identified that inflammatory and extracellular matrix integrity pathways associated with AMD pathogenesis were both modulated by exercise, with the gene expression profiles of these modules trending towards those of healthy control retinas. Taken together this study indicates that voluntary exercise may confer retinal protection against degeneration and drives the transcriptomic profile of the retina more towards what is observed in a healthy phenotype.

## Methods

### Animal paradigms

#### Animal handling

All experiments were conducted in accordance with the ARVO Statement for the Use of Animals in Ophthalmic and Vision Research and with approval from the Australian National University’s (ANU) Animal Experimentation Ethics Committee (AEEC) (Ethics ID: A2020/41; Rodent models and treatments for retinal degenerations). Adult male and female C57BL/6J wild-type (WT) mice (aged 50 postnatal days; (P50) at experimental onset) were purchased from Australian BioResources (ABR), (Moss Vale, New South Wales, Australia). Mice were bred, reared and housed under 12 h light/dark cycle conditions (5 lux) with free access to food and water.

#### Voluntary exercise model

Mice were housed individually in standard type III rodent cages with, or without TSE Systems running wheels (drum diameter of 115 mm and width of 40 mm, Cat# E−303400-RW-V-M-D1-S) for 14 or 28 days. Mice were single-housed and acclimatised to their boxes/running wheels for 3 days prior to experimental onset. Mice housed with running wheels (exercise) had unlimited and free access to wheels, while control mice (sedentary) were housed in cages of the same dimensions with no running wheels but chew block and half-tunnel enrichment. It should be noted that sedentary mice are simply those that did not have access to a running wheel and there was nothing preventing them from moving within their cages. All running wheel drums were connected to a basic unit (TSE Systems, Cat# 303400-RW-V-M-BU) for each cage. All units were connected to a central control unit (TSE Systems, Cat# E−303400-C/16) for running measurements on the PhemoMaster Control System software. For exercised mice, data on total distance (km), total time (mins), total number of runs, max speed (m/s) and average speed (m/s) were collected every hour and collated at the end of each day and on experiment completion. Data on spontaneous in-cage physical activity performed by mice housed without running wheels was obtained from previous work published by Pernold et al. (2019). This study employed both capacitance sensing technology (CST) and video tracking to examine the movement of C57BL/6J mice housed in similarly sized cages (Pernold et al., 2019). All mice were housed under 12 h light/dark cycle conditions (5 lux), with free access to food, water and environmental enrichment in the form of chew blocks.

#### Photo-oxidative damage

Exercised and sedentary mice were subjected to photo-oxidative damage (PD) for 5 days as described previously (Natoli et al., 2016). Briefly, animals were placed into Perspex boxes coated with a reflective interior surface and exposed to 100 K lux white light from light-emitting diodes (LED, High CRI LED, Yuji, Beijing). The LED is a 100-W 65,000 k natural white LED with an emission spectrum more closely resembling daylight than halogen or incandescent bulbs. No exercise wheels were provided in these PD boxes. Animals were administered pupil dilator (Minims® atropine sulphate 1% w/v; Bausch and Lomb) to both eyes twice a day during the course of the damage paradigm. Following degeneration paradigms, retinal function and morphology were assessed and compared between exercised and sedentary mice.

### Retinal assessment

#### Retinal function via electroretinography (ERG)

To assess retinal function full-field scotopic ERG was performed as previously described (Natoli et al., 2016). Briefly, mice were dark-adapted overnight before being anesthetized with an intraperitoneal injection of Ketamine (100 mg/kg; Troy Laboratories, NSW, Australia) and Xylazil (10 mg/kg; Troy Laboratories, NSW, Australia). Both pupils were dilated with one drop each of 2.5% w/v Phenylephrine hydrochloride and 1% w/v Tropicamide (Bausch and Lomb, NY, United States).

Anesthetized and pupil-dilated mice were placed on the thermally regulated stage of the Celeris ERG system (Diagnosys LLC, MA, United States). The Celeris ERG system has combined Ag/AgCl electrode-stimulator eye probes that measure the response from both eyes simultaneously and uses 32-bit ultra-low noise amplifiers fitted with impedance testing. Eye probes were cleaned with 70% ethanol and then a 0.3% Hypromellose eye drop solution (GenTeal; Novartis, NSW, Australia) was applied to both probes. The probes were then placed covering and just touching the surface of each eye. A single- or twin-flash paradigm was used to elicit a mixed response from rods and cones. Flash stimuli for mixed responses were provided using 6500K white flash luminance range over stimulus intensities from −0.01 – 40 log.cd.s.m^–2^. Responses were recorded and analysed using Espion V6 Software (Diagnosys LLC, MA, United States). Statistics were performed in Prism V7.0 using a two-way analysis of variance (ANOVA) to test for differences in a-wave and b-wave responses. Data was expressed as the mean wave amplitude ± SEM (μV).

#### Optical coherence tomography (OCT)

Cross-sectional and fundus images of live mouse retinas were taken using a MICRON® IV device (Phoenix-Micron, Inc., OR, United States). Cross-sectional images were taken at 1 mm increments from the optic nerve. Eye gel (GenTeal; Novartis, NSW, Australia) was administered to both eyes for recovery. Using OCT cross-sectional retinal images, and ImageJ V2.0 software (National Institutes of Health, Bethesda, MD, United States), the thickness of the outer nuclear layer (ONL), was calculated as the ratio to the whole retinal thickness (outer limiting membrane to the inner limiting membrane).

### Retinal tissue analysis

#### Tissue collection and preparation

Animals were ethically euthanized with CO_2_ following ERG/OCT analysis. The superior surface of the left eye was marked and enucleated, then immersed in 4% paraformaldehyde for 3 h. Eyes were then cryopreserved in 15% sucrose solution overnight, embedded in OCT medium (Tissue Tek, Sakura, Japan) and cryosectioned at 12 μm in a parasagittal plane (superior to inferior) using a CM 1850 Cryostat (Leica Biosystems, Germany). To ensure accurate comparisons were made for histological analysis, only sections containing the optic nerve head were used for analysis. The retina from the right eye was excised through a corneal incision and placed into RNAlater solution (Thermo Fisher Scientific, MA, United States) at 4°C overnight and then stored at −80°C until further use.

#### Immunolabelling

Immunohistochemical analysis of retinal cryosections was performed as previously described (Rutar et al., 2015). Fluorescence was visualized and images taken using a laser-scanning A1^+^ confocal microscope at ×20 and ×40 magnification (Nikon, Tokyo, Japan). Images panels were analyzed using ImageJ V2.0 software and assembled using Illustrator software (Adobe Systems, CA, United States).

#### IBA-1 immunohistochemistry

Immunolabeling for IBA-1 (1:500, 019-19741, Wako, Osaka, Japan) and quantification was performed as previously described (Rutar et al., 2015). The number of IBA-1^+^ cells (a marker of retinal microglia and macrophages) was counted across the superior and inferior retina using two retinal sections per mouse and then averaged. Retinal cryosections were stained with the DNA-specific dye bisbenzimide (1:10,000, Sigma-Aldrich, MO, United States) to visualise the cellular layers.

#### TUNEL assay

Terminal deoxynucleotidyl transferase (TdT) dUTP nick end labeling (TUNEL), was used as a measure of photoreceptor cell death. TUNEL *in situ* labeling was performed on retinal cryosections using a Tdt enzyme (Cat# 3333566001, Sigma-Aldrich, MO, United States) and biotinylated deoxyuridine triphosphate (dUTP) (Cat# 11093070910, Sigma-Aldrich, MO, United States) as previously described (Natoli et al., 2010). Images of TUNEL staining were captured with the A1^+^Nikon confocal microscope at ×20 and ×40 magnification. The total number of TUNEL^+^ cells were counted including both the superior and inferior retina using two retinal sections along the entire arc of the retina per animal and is represented as the average number of TUNEL^+^ cells per retinal section.

To further quantify photoreceptor survival, the thickness of the ONL on retinal cryosections was determined by counting the number of nuclei rows (photoreceptor cell bodies) in the area of retinal lesion development (1 mm superior to the optic nerve head). Photoreceptor cell row quantification was performed five times per retina using two retinal cryosections at comparable locations per mouse.

### RNA sequencing

#### RNA extraction

RNA extraction was performed using RNAqueous micro total RNA isolation kit (Thermo Fisher Scientific, MA, United States) according to the manufacturer’s instructions. The concentration and purity of each RNA sample was assessed using the ND-1000 spectrophotometer (Nanodrop Technologies, DE, United States).

#### High-throughput sequencing (HTS) and bioinformatics

RNA was placed in RNA stabilisation tubes (Azenta Life Sciences, Suzhou, China) and shipped to Azenta Life Sciences (Suzhou, China) for bulk RNA sequencing.

RNA libraries were constructed with the Illumina TruSeq RNA unstranded library preparation kit using polyA selection for mRNA enrichment, then sequenced on the Illumina NovaSeq6000 platform acquiring ∼20 million, 150 base-pair, paired-end reads per sample. Read phred scores, adapter/index contamination were checked with FastQC (Babraham Bioinformatics), then aligned to mouse genome (mm39) using HISAT2 aligner with default parameters. Alignments were summarised with featureCounts, genes with low expression (<1 count per million) were filtered out, and normalisation factors for remaining genes were calculated using trimmed means of m-values (TMM) (Robinson and Oshlack, 2010) method.

### Statistical and clustering analyses

RNA sequencing normalised counts were prepared for linear modelling using *voomWithQualityWeights* transformation (Law et al., 2014) and then a statistical model was fitted using *lmFit* (Ritchie et al., 2015) and moderated t-statistics were computed using *ebayes* function. Tables of fold change and *p*-value estimates were generated with top Table function and genes with *p*-value<0.05 and absolute fold changes >0.5 were deemed differentially expressed. Principal component analysis (PCA) and hierarchical clustering (HC) were used as methods for sample clustering and computations were performed using *prcomp* and *hclust* R functions (Team RC, 2019). Gene-wise scaling was implemented prior to sample clustering and Euclidean distance (calculated with *dist* R function) and complete linkage were used as clustering distance and agglomeration method respectively. Statistical testing of sample grouping on PCA was performed using the envfit function from the vegan R package using 10^6 sample permutations. Modular gene co-expression analysis and gene set enrichment analysis were carried out using CEMiTool R package and MSigDB C2 canonical pathway as reference set (Subramanian et al., 2005; Russo et al., 2018). Unpaired Student’s *t*-tests, one-way analysis of variance (ANOVA), or two-way ANOVA were performed using Prism V7.0. *p*-values < 0.05 were deemed statistically significant. All data was expressed as the mean ± SEM. Normality and homoscedasticity assumptions for parametric tests were visually verified by inspecting distributions of standardised residuals and the relationship between the predictor variables and the square root of standardised residuals. Kruskal–Wallis and Wilcoxon rank sum tests were employed where parametric tests were deemed unsuitable.

## Results

### Voluntary exercise paradigm and daily running data outputs

**FIGURE 1:**
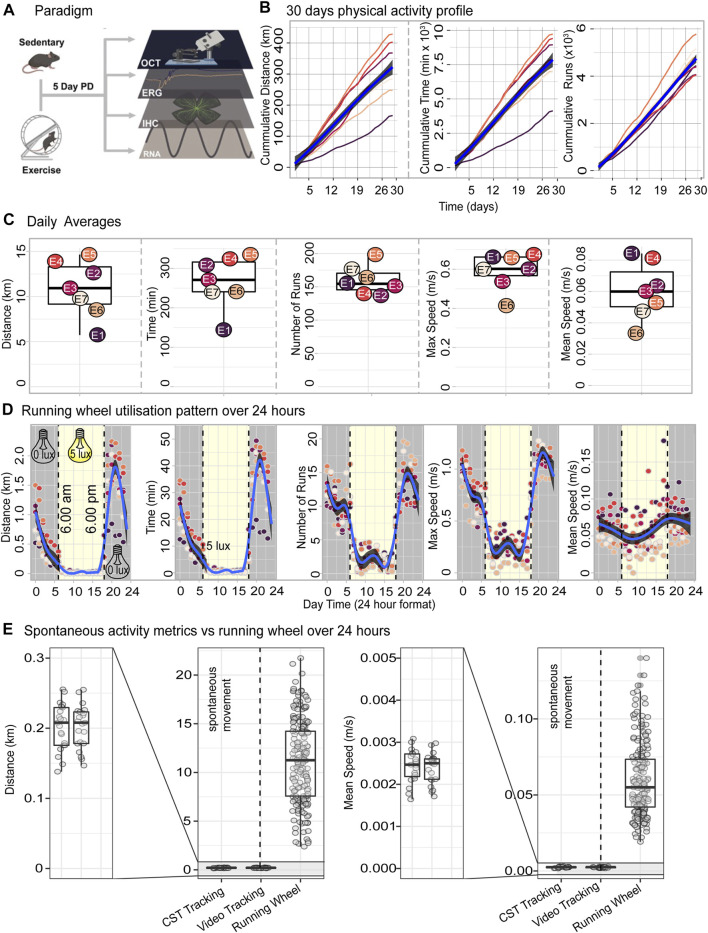
Voluntary exercise paradigm and daily running data outputs. **(A)** Schematic depicting voluntary exercise and degeneration paradigms, with output measures of retinal function, morphology and RNA sequencing. **(B)** Cumulative distance, time and runs made chronologically per animal. **(C)** Box-and-whisker graphs showing daily and average running wheel outputs: distance (km), time (mins), number of runs, max speed (m/s) and, average speed (m/s) across 28 days for each animal. Two days were omitted due to incomplete data recordings from required cage maintenance. **(D)** Utilisation of running wheel during the 12 h light/dark housing conditions. Blue lines show the trend in physical activity over 24 h summarised using a generalised additive model. Grey shaded area indicates the 95% confidence interval. **(E)** Daily distance and mean speed resulting from access to the running wheels or spontaneous in-cage movement. *n* = 7.

Exercise has well known and documented systemic benefits, including to the CNS and in protection against neurodegenerative diseases (reviewed in Chu-Tan et al. (2022). Therefore, to investigate the effects of exercise on retinal protection against degeneration, mice either with or without (sedentary) free-access to running wheels for 14 or 28 days (14 days in supplementary data), were subjected to PD-induced retinal degeneration. Note that for the 14-day experiments, mixtures of male and female mice were used due to experimental constraints. Therefore, this may not be directly comparable to the 28-day data, where only females were used to control for sex-associated effects. We thus focus on the 28-day cohorts, with the 14-day experiment placed in the supplementary data and alluded to. Following experimental paradigms, retinas were analysed and compared between groups for functional, morphological and molecular changes (Figure 1A). To verify exercise outputs, and correlate running data to measures of retinal protection; recorded measures of distance (km), time (min), number of runs, max speed (m/s) and, average speed (m/s) in hour bins over a period of 28 days were analysed.

Mice initiated physical activity immediately after being provided with access to the running wheel and continued to utilise the running wheel until the cessation of the 28-day exercise period (Figure 1B). After deriving daily totals for distance, time, and number of runs and deriving cumulative sums over the 28 days period we report that, mice on average ran a total distance of 316 km (166—428, SEM 32.3), utilised the running wheel for an average of 128 h (70—128, SEM 677) and performed on average a total of 4,670 individual runs (4,048—5,799, SEM 220) (Figure 1B). All three exercise parameters were accrued over the 28 days in a consistent linear fashion with an almost invariant slope (Figure 1B). To understand the exercise behaviour of mice in further detail, daily averages were next computed for distance, time, number of runs as well as mean and maximum speed. Mice on average, ran 10.2 km (5.54—14.2, SE 1.84), 257 min (137—323, SE 24.3), and 155 runs (134—192, SE7.9) runs per day, with a max speed of 0.598 m/s (0.417—0.679, SE 0.035), and average speed of 0.060 m/s (0.033—0.083, SE 0.0068) (Figure 1C). Intraday exercise activity as measured by running distance, time, number of runs and maximum speed occurred cyclically and coincided with the 12 h light/dark housing conditions with most of the exercise activity being performed during the nocturnal phase (Figure 1D).

Next, we compared the daily exercise output of mice with access to running wheels to that of mice performing spontaneous in-cage activity without running wheels as reported by Pernold et al. (2019). The metrics chosen for this comparison were daily distance and daily mean speed measured using both CST and video tracking by Pernold et al. (2019). In the absence of a running wheel, mice covered a distance of 0.2 km per day at an average speed of 0.0024 m/s (Figure 1E) which represents approximately 50-fold decrease in activity level when compared to the 10.2 km daily distance and 0.060 m/s daily mean speed registered on the running wheel system (Figure 1E).

Taken together, tracking of the exercise output over a 28-day period demonstrates that the use of a running wheel setup is a reliable method for increasing physical activity in mice, orders of magnitude over the level of activity performed as a result of spontaneous in-cage movement.

### Voluntary exercise protects retinal function and morphology against degeneration

**FIGURE 2:**
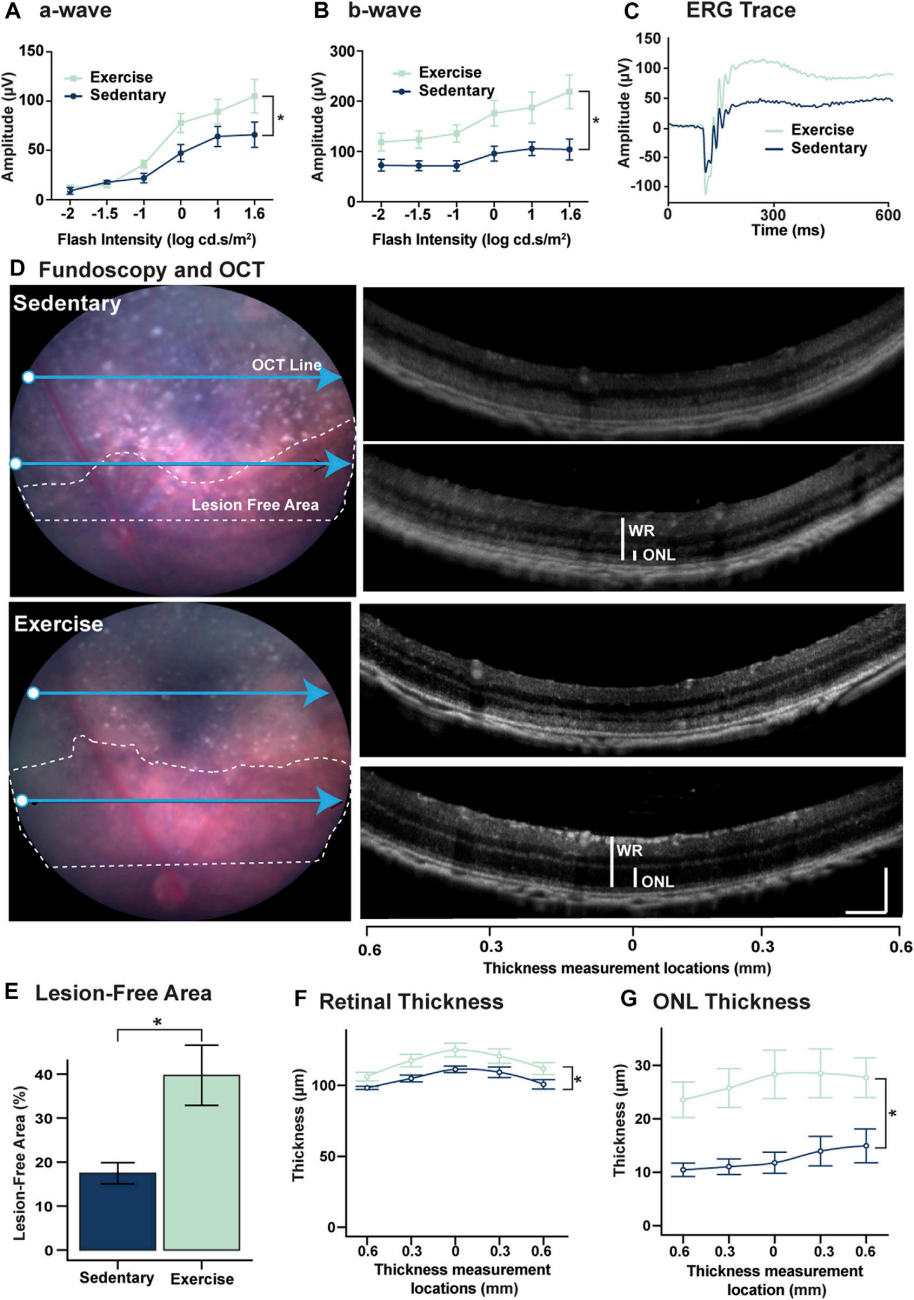
Voluntary exercise protects retinal function and morphology against degeneration. **(A–C)** Mice with access to voluntary exercise had significantly improved retinal function in both **(A)** a-wave, and **(B)** b-wave measures. **(C)** Representative ERG trace of exercised and sedentary mice using a flash intensity of 1.6 log cd.s.m^2^. **(D)** Representative fundi from exercise and sedentary mice. White dashed lines show retinal areas free of severe degeneration marked by the presence of hyper-reflective puncta and hyperpigmentation. Blue arrows show the corresponding location of the OCT retinas on the right. **(E)** Quantification of lesion free areas expressed as a percent of the total retinal area superior to the optic nerve head. **(F)** Quantification of total retinal thickness and **(G)** ONL thickness. OCT images used for thickness measurements were captured at two-disc diameters superior to the optic nerve head measurements were taken directly above the optic nerve head and at 0.3 and 0.6 mm nasally and temporally. *Significance using two-way ANOVA with Sidak’s *post hoc* test for multiple comparisons, or Student's *t*-test, *p* < 0.05 and error bars indicate SEM, *n* = 9.

To investigate the effect of exercise on retinal protection against degeneration, mice (n = 8–9) were subjected to 5 days of photo-oxidative damage immediately after 30 days of exercise then retinal function and morphology were assessed using ERG, OCT and fundoscopy. Relative to sedentary controls, exercised mice had significantly improved retinal function for both a-wave (photoreceptor function) and b-wave (second order neuron) (*p* < 0.05, Figures 2A–C). Fundus/OCT imaging (Figures 2D–G), further demonstrate the protective effects of exercise on the retina during degeneration. Areas of free of degenerative lesions (defines as retinal regions presenting with of hyperreflective puncta, hyperpigmentation and severe ONL loss shown by dashed areas in Figure 2D) were larger in exercised mice (39.7% ± 6.84%) compared to sedentary controls (17.4% ± 2.42%) (Figure 2E, *p* < 0.05). Thickness measurements carried out two-disc diameters superior to the optic nerve head indicated that both the ONL (Figure 2E) and whole retinal thickness (WR) (Figure 2F) were significantly (*p* < 0.05) larger in the exercise animals across five distinct eccentricities. Exercised mice had a WR of 116.2 ± 2.08 μm and ONL thickness of 26.7 ± 1.73 μm while the same two measurements were 104.8 ± 1.34 μm and 12.4 ± 0.99 μm for sedentary mice respectively. In the 14-day cohort, no significant differences were observed in either ERG or ONL thickness (Supplementary Figure S1).

### Voluntary exercise protects the retina against photoreceptor cell death and inflammation

**FIGURE 3:**
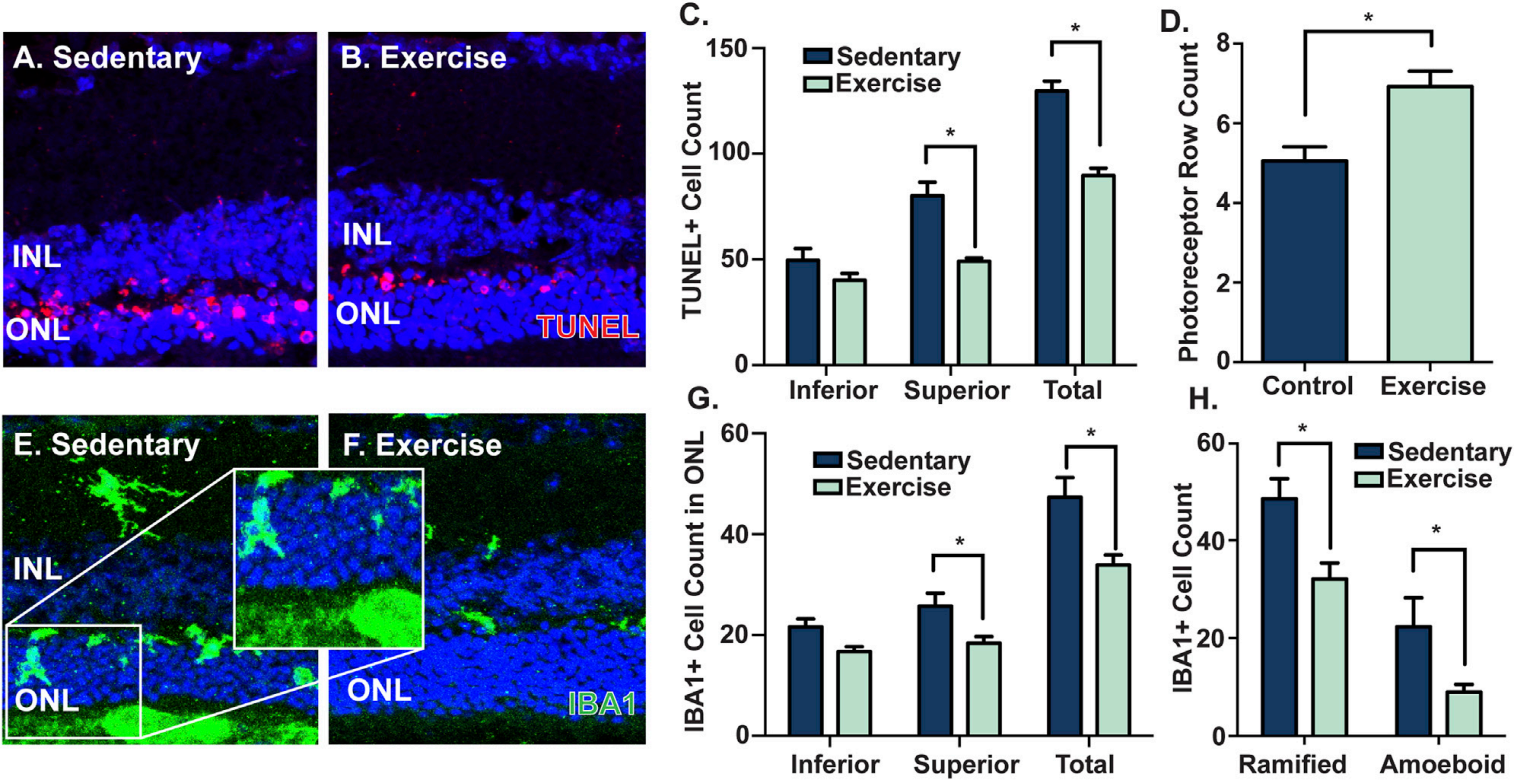
Voluntary exercise protects the retina against photoreceptor cell death and inflammation. Mice with access to voluntary exercise had decreased levels of photoreceptor cell death, as shown in **(A–B)** representative confocal images and quantified by **(C)** reduced numbers of total and superior TUNEL^+^ cells (red) in the ONL, and **(D)** increased numbers of photoreceptor nuclei rows, compared to sedentary controls. Further, exercised mice had reduced levels of IBA1^+^ cells (green) in the ONL, as shown by **(E–F)** representative confocal images, and quantified by **(G–H)** decreased numbers of total, superior, ramified and amoeboid IBA1^+^ cells in the ONL. *Significance using a Student's *t*-test, *p* < 0.05 and error bars indicate SEM. Scale bar = 50 μM. *n* = 9.

Given the partial protection in retinal function and preservation of retinal morphology observed in exercised mice, (Figure 2), key features of retinal degeneration (photoreceptor cell death, and presence and activation of microglia/macrophage immune cells) were measured and compared between sedentary and exercised mice following PD. Compared to sedentary controls, mice with access to voluntary exercise had significantly reduced levels of photoreceptor cell death (*p* < 0.05, Figures 3A,B), as measured by a decreased number of TUNEL^+^ cells across the ONL, and in the superior ONL (*p* < 0.05, Figure 3C), and an increased number of photoreceptor rows (*p* < 0.05, Figure 3D). Further, as a measure of retinal inflammation, IBA1^+^ microglial/macrophage cells were counted and morphologically characterised in the ONL. In exercised mice, IBA1^+^ cells were significantly reduced in the ONL compared to respective sedentary controls (*p* < 0.05, Figures 3E,F), notably with significant reductions in the superior retina (*p* < 0.05, Figure 3G), and in both ramified and amoeboid IBA1^+^ morphologies (*p* < 0.05, Figure 3H). This was true also for the 14-day cohort where photoreceptor cell death and inflammation as measured by IBA1 was significantly reduced in the exercised mice (Supplementary Figure S2). Collectively these results demonstrate the protective effect of voluntary exercise against retinal degeneration within the 28-day time frame, with functional preservation attributed to reduced levels of photoreceptor cell death and retinal inflammation in exercised mice.

### Voluntary exercise induces gene expression profile similarities to healthy control mice

**FIGURE 4:**
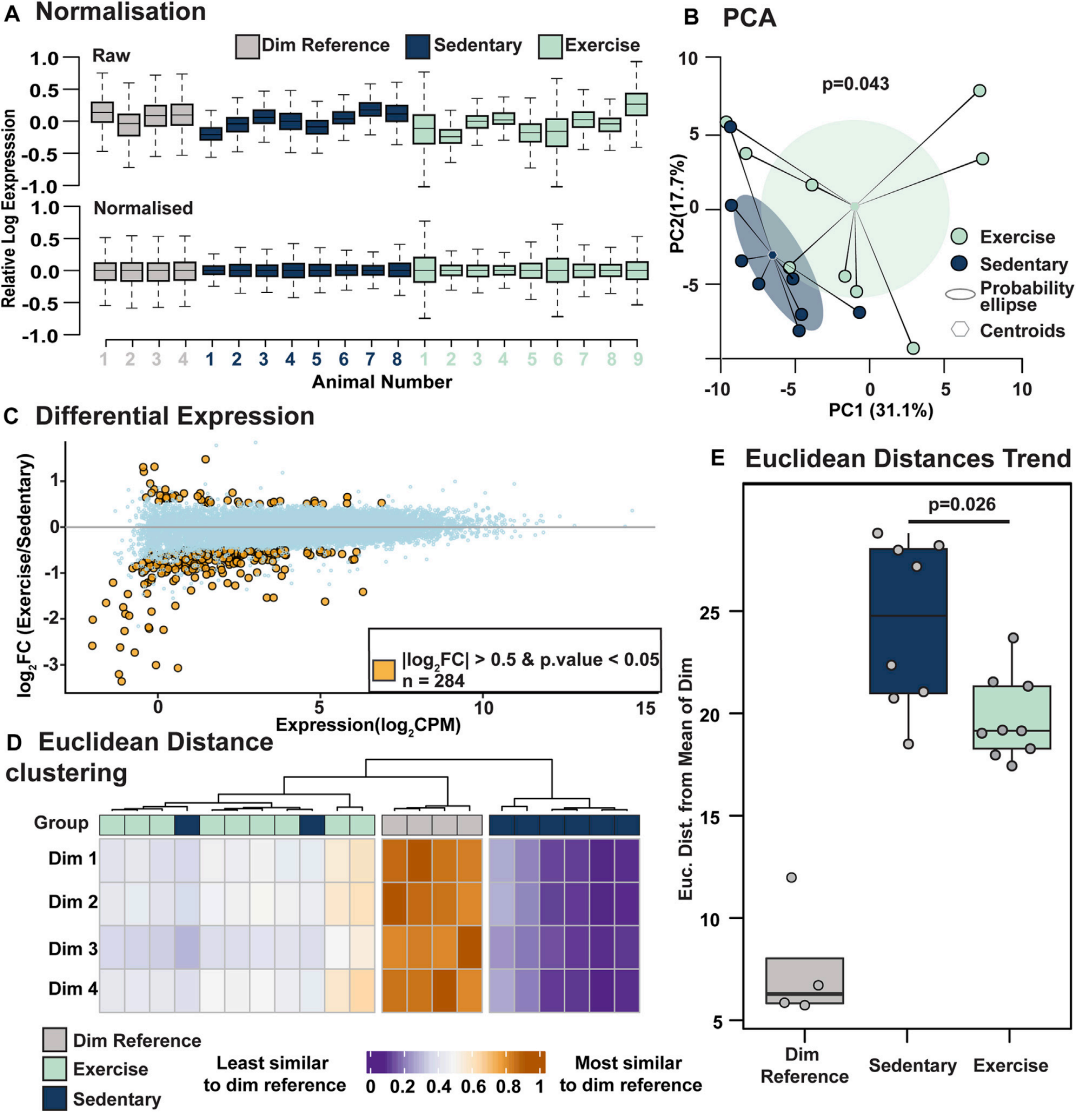
Voluntary exercise induces gene expression to a profile more similar to healthy control mice. **(A)** Relative Log Expression plots showing effective normalisation. **(B)** PCA plot showing significant clustering of the exercise and control groups. *p*-values were derived by performing *envfit* analysis on the PCA object with 10^6^ permutations. Shaded area represents the probability ellipse of each group calculated by using the group standard deviation and a confidence limit of 0.95. **(C)** Volcano plot showing differentially expressed genes between exercised and sedentary retinas (absolute *Log2FC>0.5, p < 0.05*). **(D)** Clustering of Euclidean distances calculated using expression values of differentially expressed genes. Distances for both exercise and sedentary groups were calculated relative to a reference matrix containing expression values of differentially expressed genes (exercise vs. sedentary) in heathy dim-reared retinas. This heatmap indicates that the expression values of the 284 differentially expressed genes trend more strongly towards heathy controls (dim-reared mice) values in exercised mice than in sedentary mice. **(E)** Summary of Euclidean distances between exercise and sedentary mice relative to dim-reared mice based on the 284 differentially expressed genes in **(C)**. Significance tested using Wilcoxon signed-rank test. *n = 4-9*.

To investigate the molecular pathways underpinning the observed retinal protection, bulk RNA sequencing was performed on retinal lysates from exercised and sedentary mice exposed to PD and compared to dim sedentary mice. Following read count normalisation (Figure 4A), a two-dimensional principal component analysis was employed to visualise global differences between the expression of retinal mRNA profiles from exercised and sedentary mice (Figure 4B). Using *envfit* analysis and permutation-based statistical testing, we determined that exercise and sedentary mice are arranged in two distinct clusters on the PCA space (*p* < 0.05), thus suggesting that when compared to sedentary mice, exercise induces consistent gene expression changes in mice exposed to PD (Figure 4B). Next, we sought to determine if global gene expression patterns in exercised mice shifts the RNA profile of a heathy (non-PD) mouse retina. To achieve, a subset of genes (differentially expressed between exercised and sedentary mice, log_2_fold change (FC) > 0.5, *p* < 0.05) were identified (Figure 4C; orange dots), then using the expression values of this subset of genes, the Euclidean distances (ED) of both exercise and sedentary mice to dim controls were calculated. Hierarchical clustering of EDs computed based on differentially expressed genes, indicated that 7/9 mice had a gene expression profile more similar to a heathy retina than sedentary controls (Figure 4D). Besides clustering, the magnitude of EDs was statistically tested (Figure 4E) and revealed that the exercise shortens the separation between heathy controls and PD-retinas (ED_dim-sedentary_ = 24.5 ± 0.47, ED_dim-active_ = 20.2 ± 0.21, *p* < 0.05). Taken together these results suggest that exercise partially shifts the transcriptome of the PD retina towards an expression profile that aligns more closely to a dim-reared, “healthy” retina.

### Modular gene co-expression analysis identifies inflammatory and extracellular matrix integrity pathway modulation in response to voluntary exercise

**FIGURE 5:**
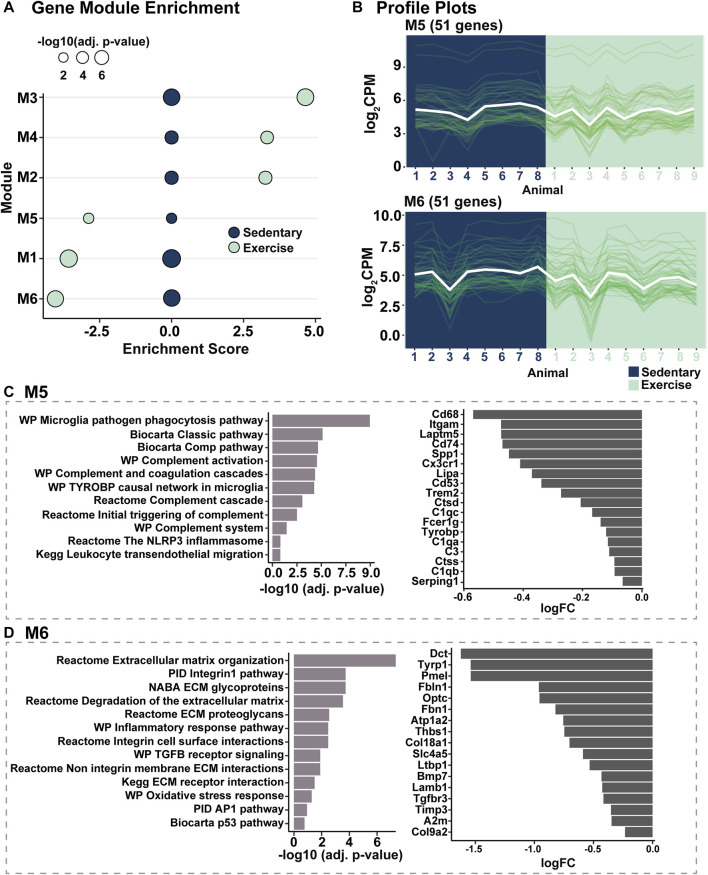
Modular gene co-expression analysis identifies inflammatory and extracellular matrix integrity pathway modulation in response to voluntary exercise. **(A)** Gene module enrichment identified six modules (M) which were significantly enriched (M2-M4, positively enriched, M1, M5 and M6, decreased enrichment) compared to sedentary controls. **(B)** Profile plots of genes within modules M5 and M6 to show representative similar gene expression trends within modules. **(C,D)** Pathway analyses showing inflammatory and extracellular matrix integrity pathways were associated with genes in M5 and M6 respectively.

As the retinal gene expression profile from exercised mice was found to be trending towards that of healthy control (dim) retinas, to identify the specific gene and gene pathways modulated in response to voluntary exercise and responsible for this profile shift, modular gene co-expression analysis was performed in combination with gene set enrichment analysis. Following modularisation of normalised gene counts, six gene expression modules were identified, with modules (M) M2, M3, and M4 showing significantly positive enrichment, and modules M1, M5 and M6 showing significantly decreased enrichment in exercised mice compared to sedentary controls (*p* < 0.05, Figure 5A). Of the six modules (Supplementary Table S2), M5 and M6 (Figure 5B) were further explored because they contained signalling pathways and immune mediators central to the pathogenesis of retinal degeneration. Pathways associated M5 included microglial and leukocyte cell migration and phagocytosis, complement activation and inflammasome activation (*p* < 0.05, Figure 5C). Consistent with a downregulation of in the enrichment of pathways in M5, key mediators of retinal inflammation such as *C3, C1q, Cx3CR1 and Cd74* were observed to be downregulated in exercised animals (Figure 5C) relative to sedentary controls. Further, pathways significantly associated with M6 genes, and known to play key roles in retinal degeneration included extracellular matrix modulation, integrity and organisation, and oxidative stress responses (*p* < 0.05, Figure 5D).

Taken together, these results support a role for exercise-mediated retinal protection against degeneration through a combination of influencing inflammation and ECM modulation and show a transcriptomic shift towards a healthy phenotype.

## Discussion

The benefit exercise provides to the CNS are only just starting to be discovered, with insight into the molecular changes that occur, in particular in the retina, still largely unknown. With accumulating evidence demonstrating the ability of physical activity to surmount neuro- and retinal degenerative pathophysiology (reviewed in Chu-Tan et al. (2022), there is a critical need to unlock the complete molecular events underpinning exercise-induced neuroprotection. Therefore, in this work, using a voluntary model of aerobic exercise that allows for free access to open running wheels and a well-characterised degeneration model that recapitulates key facets of AMD pathogenesis, we provide an overview of the molecular changes attributed to voluntary exercise in the retina, and how they may confer protection against retinal degeneration. Our results demonstrated that voluntary exercise in mice for 28 days prior to retinal degeneration provided significant protection to retinal function and integrity, and reduced levels of photoreceptor cell death and microglial/macrophage activation/infiltration. Further, that this observed protection may be attributable to decreased activation of inflammatory pathways and preservation of extracellular matrix integrity as hyper-inflammatory responses and loss of ECM are implicated in retinal degeneration and AMD progression. Overall, our results indicated that exercise was able to induce a transcriptomic gene expression profile shift towards that of a healthy retina, ultimately supporting the potential use of exercise as a non-pharmacological intervention to slow the progression of retinal degenerations and AMD, and identification of a novel therapeutic factors that can target key pathways such as complement.

### Exercise induces molecular changes that drive towards a healthy retinal phenotype

We have provided for the first time a foundational picture of the major molecular pathways underlying exercise-mediated retinal protection and identified two major themes that had overall transcriptomic shifts towards a healthy phenotype: inflammation, and extracellular matrix integrity.

Over-activation of the immune response within the CNS is a hallmark feature of neurodegeneration and has been established to influence the progression of retinal degenerations, including AMD (Ambati et al., 2013; Kauppinen et al., 2016). Chronic and progressive inflammation in retinal degenerations is primarily mediated by the innate immune system, and largely controlled by both the resident retinal microglia as well as recruited peripheral macrophages (Amor et al., 2010; Ambati et al., 2013; Kauppinen et al., 2016). Reducing pathological levels of inflammation may therefore be a key therapeutic strategy in slowing the progression of retinal degenerations, making the anti-inflammatory properties of exercise a considerable therapeutic interest. There is a strong link between exercise and the inflammatory system in the human body including in the CNS (Nieman and Wentz, 2019). Exercise is known to modulate key inflammatory mechanisms, mitigating and regulating systemic inflammation in neurological diseases such as multiple sclerosis (Dalgas et al., 2012), systemic lupus erythematous (Perandini et al., 2012), Alzheimer’s and Parkinson’s diseases (Fuller et al., 2020), and preclinical models of retinal degeneration (Crowston et al., 2017; Sellers et al., 2019; Zhang et al., 2019; Dantis Pereira de Campos et al., 2020). However, these anti-neuroinflammatory effects, whilst observed, have not been well-substantiated or often investigated past single target identification.

Results from this study support the anti-inflammatory effects of exercise, with reduced infiltration and activation of IBA1^+^ microglia/macrophages in the retina of exercised mice. in particular, reduced level of microglia/macrophages were detected in the ONL of the central/superior retina—an area well-characterised to be the primary site of damage during light-induced degeneration (Natoli et al., 2016). In support of these observations, pathway analysis of RNA sequencing results indicated decreased expression of microglial/macrophage markers, including *Cd68*, *Cd74* and *Cx3Cr1*—all known to have increased expression and drive pathological inflammatory processes in AMD (Ambati et al., 2013). Further, bioinformatic analyses indicated that the complement system, which has been previously identified as a major instigator of inflammatory dysregulation in AMD (Edwards et al., 2005; Hageman et al., 2005; Haines et al., 2005; Klein et al., 2005), was less active in exercised animals. Notably, complement pathway genes *C1qa, C1qb, C1qc, C3* and *Serping1* were all significantly downregulated in the retinas of exercised mice. In addition, the *Nlrp3* pathway also appeared to be less active in exercised animals. In AMD, NLRP3-dependent cleavage and activation of pro-inflammatory cytokine IL-1β (Halle et al., 2008) has been shown to drastically increase excessive inflammation in the retina, (Wooff et al., 2019), and is identified to play a key role in disease pathogenesis causing progressive photoreceptor cell death. Taken together, these results highlight potential pathways modulated in response to exercise that could be key to dampening the observed retinal immune response and providing protection against degeneration.

In addition to inflammatory modulation, key findings from this work identified that extracellular matrix integrity pathways were also significantly modulated in response to exercise. The extracellular matrix, acellular protein-rich scaffold, consisting of proteoglycans and glycoproteins, plays an important homeostatic role in maintaining retinal health and integrity functioning in both structural and mechanical support (Al-Ubaidi et al., 2013). In retinal degenerations including AMD, extracellular matrix degradation and remodelling have been strongly associated with disease pathogenesis (Al-Ubaidi et al., 2013). Results from our study show that in exercised mice extracellular matrix genes such as *Itgam* (*Cd11b*)*,* a key mediator of monocyte adhesion (Solovjov et al., 2005), were significantly downregulated compared to sedentary mice. As integrin-mediated adhesion to the ECM plays a key role in infiltration of immune cells during damage or degeneration (Baiula et al., 2021; Mrugacz et al., 2021), modulations in this pathway may lead to reduced immune cell presence in the retina hereby haltering damaging and pathological immune cascades. Our results strongly support this hypothesis, with an observed reduction in recruited IBA1^+^ cells and microglial/macrophage markers, as well as a downregulation in several notable immune pathways known to be propagated by these immune cells and involved in AMD disease progression (Rutar et al., 2015; Jiao et al., 2018). Additionally, Tissue Inhibitor of Metalloproteinases 3 (*Timp3*) which was also found to be downregulated in exercised mice, has been heavily implicated as a potential candidate in AMD pathogenesis, with elevated levels seen to contribute to the thickening of Bruch’s membrane commonly seen in AMD patients (Kamei and Hollyfield, 1999). The diverse roles of *Timp3* in inflammation, angiogenesis, and extracellular matrix turnover/degradation, have made it a closely looked at molecule for complex degenerative diseases including Alzheimer’s disease and AMD (Dewing et al., 2019).

These novel results provide further insight into the molecular pathways that are involved in providing neuronal protection to the retina following exercise. It must be noted that unlike in previous studies (Poo, 2001; Binder and Scharfman, 2004), we did not observe a significant change in brain-derived neurotrophic factor (BDNF) which has been a heavily postulated to be a crucial molecule in exercise-mediated neuronal protection (Poo, 2001; Binder and Scharfman, 2004). However, the sensitive nature of *Bdnf* deems it difficult to detect changes without conducting high-sensitivity experiments on its effect on specific pathways such as TrkB (Allen et al., 2018) or detecting their protein rather than RNA levels. Further, the protective effects of exercise are pleiotropic with the same gene influencing different phenotypic traits and expression patterns differing based on various forms of exercise (Côté et al., 2011; Keeler et al., 2012). Therefore, we cannot rule out a possibility of *Bdnf* playing a role in the protection that we observed despite no significant changes to it or its primary receptor.

### Voluntary exercise is protective against retinal degeneration

To date, the majority of animal studies investigating the effect of exercise in the retina have used forced exercise models where an often unpleasant or painful stimulus is used to ensure the animals complete a defined amount of physical activity (Ji et al., 2013; Kim et al., 2013; Chrysostomou et al., 2014; Lawson et al., 2014; Chrysostomou et al., 2016; Allen et al., 2018; Mees et al., 2019; Dantis Pereira de Campos et al., 2020). While it is understandable that studies using forced-exercise models use this method in an attempt to ensure equal and consistent levels of desired exercise, output measures from our study revealed that mice would voluntarily and consistently utilize the running wheels, running large distances of 10.2 km/day on average, mitigating any need to force this seemingly natural behavior onto them. Further, given the stress and anxiety-like behaviours known to be inflicted in these forced exercise models (Leasure and Jones, 2008), forced-exercise methods may potential confound the true effects of exercise. Studies have in fact demonstrated not only significant differences in brain behaviours, but also differences in the intensity and duration of activity under forced and voluntary exercise animal paradigms, despite attempts at standardization (Leasure and Jones, 2008). Even when distance was held constant, voluntary exercisers reached much higher speeds but for shorter time periods (43.7 m/min in voluntary exercise animals and 15.5 m/min in forced exercise animals) (Leasure and Jones, 2008). Due to the much higher speeds in the voluntary exercise groups, to effectively standardise the distance; forced exercise animals had to run for far-longer periods of time, creating inherent differences within the exercise paradigms (Leasure and Jones, 2008), and making the results difficult to compare and decipher. Further, stress caused by such models may disturb the inflammatory balance of the retina and impact on output measures as a result (Malan et al., 2020). As the use and output measures of voluntary models of exercise more closely mimic what may be prescribed in a clinical setting to human patients, it appears more intuitive to use voluntary models such as the one in this study to truly understand the molecular mechanisms and pathways underlying exercise-induced neuroprotection.

It should also be noted that our supplementary results demonstrated that there at 14 days of exercise, the protective effect seen at 28 days is not present in the functional results and only in histology did we observe protection against photo-oxidative damage. Whilst interesting and potentially indicative of a certain amount of exercise needed to provide the full suite of protection, these results cannot be directly compared due to differences in sex and age of animals for the experiments.

### Future directions

It remains unclear precisely how systemic effects from exercise can target the retina and confer protection against degeneration. It is likely however that tissue crosstalk is occurring to communicate the message of exercise to the rest of the body. Landmark studies have in fact identified that exercise-mediated protection may be facilitated, in part, by cellular communication via extracellular vesicles (EV) (Frühbeis et al., 2015; Whitham et al., 2018). EV (including exosomes and microvesicles) are nanosized cell-to-cell delivery vesicles which are released by nearly all cell types in the body and function to induce a biological response in recipient cells (Hessvik and Llorente, 2018) via the transfer of molecular cargo, including proteins, lipids and non-coding RNA, such as miRNA (Hessvik and Llorente, 2018). Studies have shown that following exhaustive cycle and treadmill exercise, a significant increase in EV numbers was found in the circulation immediately post exercise (Frühbeis et al., 2015; Whitham et al., 2018; Brahmer et al., 2019). Further, beneficial molecular cargo including proteins and miRNA carried within exercise-EV, have been shown to be responsible for exerting systemic neuroprotective effects, including promoting advantageous metabolic changes (Castaño et al., 2020), increasing skeletal muscle fibre growth and recovery (Wu et al., 2022), and modulating immune responses in the CNS (Frühbeis et al., 2015; Whitham et al., 2018; Vechetti et al., 2021). These findings largely support a role for EV/EV cargo in mediating exercise-induced systemic protection, suggesting that investigations into the role of EV in mediating retinal protection during exercise warrant further investigations, and should be avenues for future exploration.

## Conclusion

The benefits of exercise are far-ranging with increasing evidence demonstrating the therapeutic effects of exercise in the CNS and retina. Here we have demonstrated that voluntary exercise is protective to the retina both in function and retinal integrity, likely due to inflammatory pathway modulation and maintenance of the extracellular matrix. These results will help lay the foundation for deeper investigations into the therapeutic molecular mechanisms that exercise provides in protecting against retinal degenerations such as AMD.

## Data Availability

The datasets presented in this study can be found in the article and Supplementary Material and is accessible for download from the BioProject repository under project ID PRJNA934406.

## Glossary

ABR: Australiain Bio Resources
AEEC: Animal Experimentation Ethics Committee
AMD: Age-related Macular Degeneration
ANOVA: Analysis of Variance
ANU: Australian National University
ARVO: Association for Research in Vision and Ophthalmology
Bdnf: Brain-Derived Neurotrophic Factor
CNS: Central Nervous System
CST: Capacitance Sensing Technology
ECM: Extracellular Matrix
ED: Euclidean Distance
ERG: Electroretinogram
EV: Extracellular Vesicles
HC: Hierarchical Clustering
IL-1β: Interleukin 1-Beta
INL: Inner Nuclear Layer
km: Kilometres
LED: Light-Emitting Diode
m/s: metres per second
Min: Minutes
mm: millimetres
NLRP3: NLR Family Pyrin Domain containing 3
OCT: Optical Coherence Tomography
ONL: Outer Nuclear Layer
PCA: Principal Component Analysis
PD: Photo-oxidative Damage
RNA: Ribonucleic Acid
SEM: Standard Error of the Mean
Timp3: Tissue Inhibitor of Metalloproteinases 3
Trkb: Tropomyosin Receptor Kinase B
TUNEL: Terminal deoxynucleotidyl transferase (TdT) dUTP nick end labelling
WR: Whole Retina thickness
