# Supplementary material for: Voluntary exercise modulates pathways associated with amelioration of retinal degenerative diseases
Source: Front Physiol. 2023 Mar 10;14:1116898. doi: 10.3389/fphys.2023.1116898 (PMC10036398; doi:10.3389/fphys.2023.1116898)
Supplement: Supplementary file 1 [file DataSheet4.PDF]

Australian National University

Assoc. Prof. Riccardo Natoli
John Curtin School of Medical Research (JCSMR) &
ANU Medical School
Office Room 2.371 JCSMR
Garran Road, Acton, ACT, 2601
(T) +61 2 6125 8559
(M) 0400813132
(E) riccardo.natoli@anu.edu.au

Tuesday, 6 December 2022

Dear editors of Frontiers in Physiology,

Please consider the accompanying manuscript, ***Voluntary exercise modulates pathways associated with age-related macular degeneration***, for publication in your journal Frontiers in Physiology. This is an original study that we believe will be of interest to your readership and the research community that your journal attracts. We believe this publication will have a wide-ranging appeal to the research community as it demonstrates utility of exercise as a potential therapeutic in a neurodegenerative context using a rodent model of retinal degeneration. Further, it sheds some insight into the molecular changes occurring during exercise within the retina, which could have similar affects within the pathophysiology of other central nervous system disorders such as Alzheimer's and Parkinson's disease.

Exercise has been demonstrated to be a key non-invasive therapeutic for a number of diseases, including neurodegenerative diseases such as Parkinson's, Alzheimer's and age-related macular degeneration (AMD). However, the lack of whole transcriptomic investigation into the molecular underpinnings in the CNS has resulted in a number of existing questions within the field. In this paper we have used a voluntary model of rodent exercise with access to running wheels *ad libitum* and a model of retina degeneration to explore the molecular underpinnings of exercise induced neuroprotection. Through this, we demonstrated that exercise is protective against light-induced degeneration of the retina with exercised animals having higher retinal function and less neuronal cell death and retinal inflammation. Further, we conducted RNA sequencing and showed exercised animals had a transcriptomic shift towards a non-damaged, healthy molecular profile indicative of a significant retinal protection following exercise. Specifically, pathways associated with key inflammatory and extracellular matrix integrity were significantly regulated between exercise and sedentary animals. We believe that this study provides key insight into the pathways underpinning the effects of exercise in the retina and neurodegeneration.

Therefore, we believe this paper will be of significant interest to your readership and the scientific community at large. We hope this letter and the manuscript find you well and look forward to hearing from you in the near future.

Yours sincerely,

Riccardo Natoli BSc (Hon), PhD
ANU Medical School & John Curtin School of Medical Research
ANU College of Health and Medicine
