# Supplementary figures and images for: Voluntary exercise modulates pathways associated with amelioration of retinal degenerative diseases
Source: Front Physiol. 2023 Mar 10;14:1116898. doi: 10.3389/fphys.2023.1116898 (PMC10036398; doi:10.3389/fphys.2023.1116898)

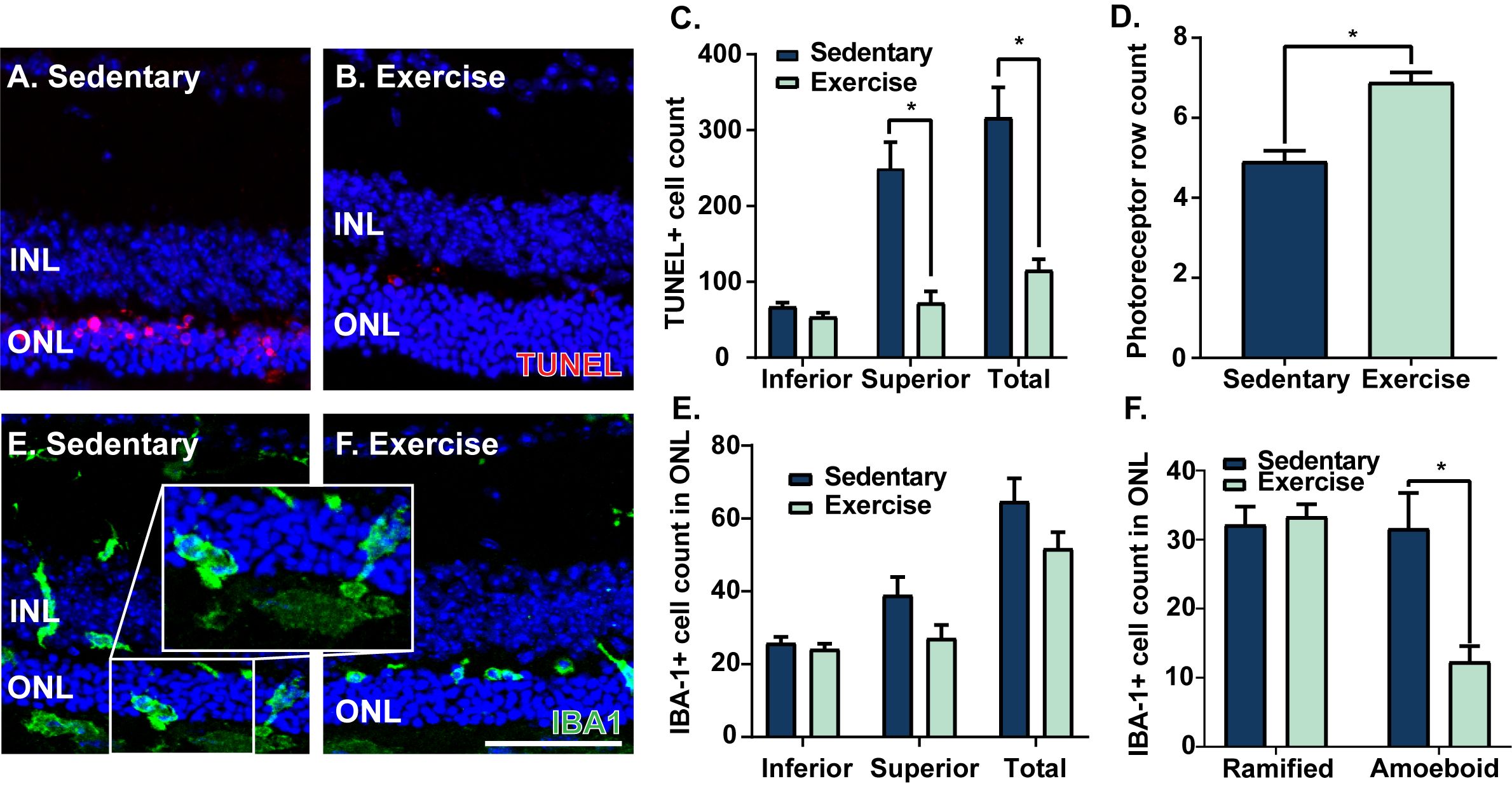

Supplement: Supplementary file 3 [file Image2.TIF]

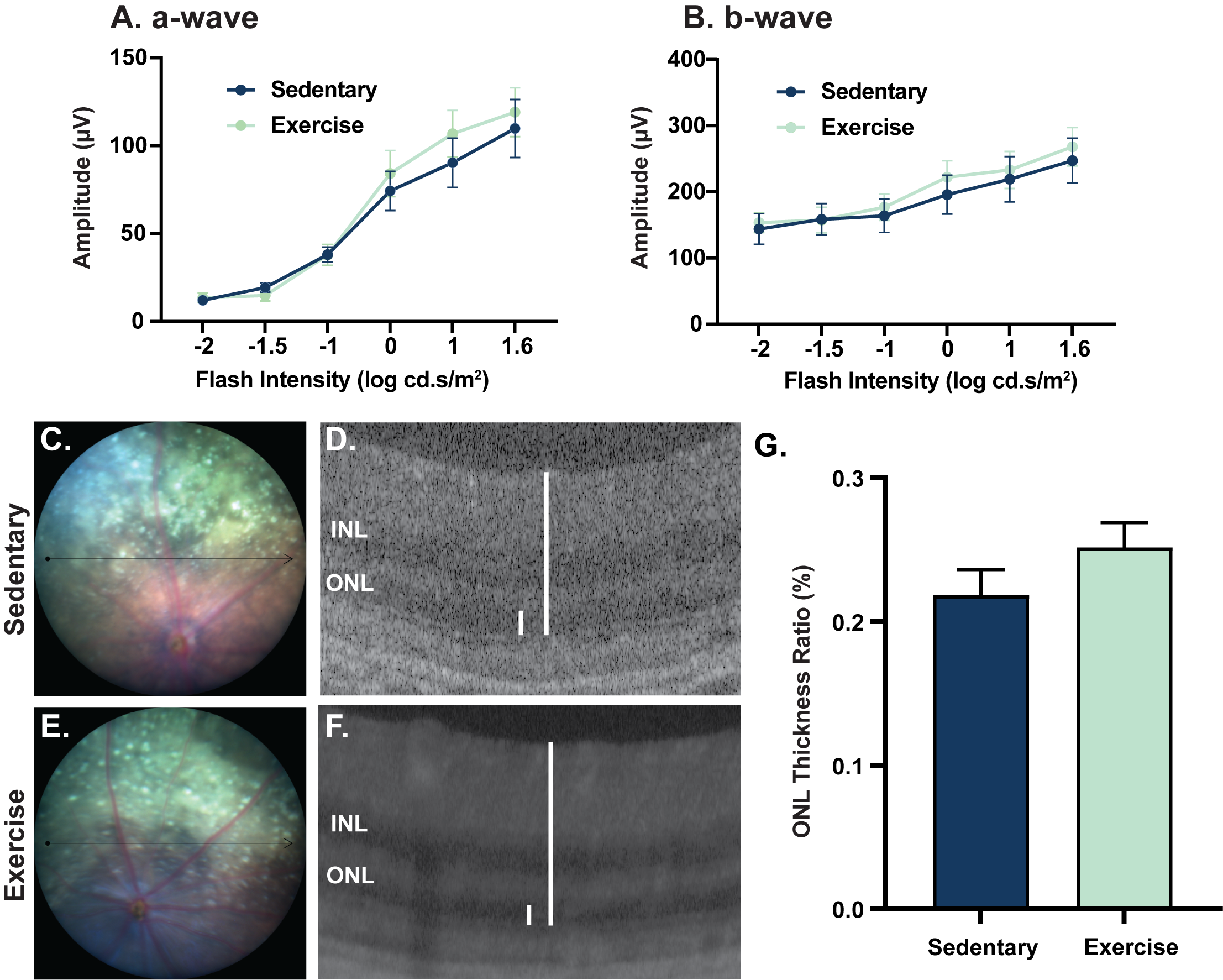

Supplement: Supplementary file 4 [file Image1.TIF]
